# Despite antiretroviral therapy (ART) rollout, most cases of tuberculosis among people with HIV in Adama, Ethiopia, occur before ART initiation

**DOI:** 10.1080/16549716.2024.2395073

**Published:** 2024-08-28

**Authors:** Patrik Bristedt, Meseker Fentie, Per Björkman, Anton Reepalu

**Affiliations:** a Clinical Infection Medicine, Department of Translational Medicine, Lund University, Malmö, Sweden; b Bacterial and Viral Diseases Research Directorate, Armauer Hansen Research Institute, Adama, Ethiopia; c Department of Infectious Diseases, Skane University Hospital, Malmö, Sweden

**Keywords:** Tuberculosis, HIV, antiretroviral treatment, Ethiopia, notifications

## Abstract

**Introduction:**

Although antiretroviral therapy (ART) leads to reduced tuberculosis (TB) incidence in people with HIV (PWH), ART recipients remain at higher risk of TB compared to HIV-seronegative people. With accelerated ART rollout in sub-Saharan Africa, increasing proportions of TB cases among PWH in people receiving long-term ART have been reported.

**Objective:**

To determine TB notifications among PWH by ART status in a mainly urban uptake area in Ethiopia during an 8-year period in connection to the introduction of the ‘test-and-treat’ strategy for HIV.

**Methods:**

PWH were identified from registers at health facilities providing ART in Adama and surrounding areas, Ethiopia 2015–2022. Annual TB notifications were compared over time. PWH within TB were categorized by ART status at the time of TB diagnosis (pre-ART TB: TB diagnosed before or ≤6 months after starting ART; ART-associated TB: TB diagnosed >6 months after starting ART).

**Results:**

Among a total of 8,926 PWH, 993 had been diagnosed with TB (11.1%); mean age 40.0 years [SD 11.8], 53.5% were men). Throughout the study period, most TB cases had been notified before ART initiation (617/993; 62.1%). ART-associated TB cases constituted a mean of 37.4% (range 23.8%–44.2%) of all TB cases among PWH annually. Median time from ART initiation to TB diagnosis among ART-associated TB was 6.0 years.

**Conclusion:**

TB notifications among PWH in this area did not decrease 2015–2022, implying persistently high risk of TB among PWH in this setting. Most TB cases occurred in ART-naïve persons, illustrating late HIV diagnosis in this population.

## Paper context

- **Main findings:** Annual numbers of TB notifications among PWH in Central Ethiopia were persistently high 2015–2022 and most cases of TB were diagnosed before or within 6 months after ART initiation, implying late diagnosis of HIV in these individuals.
- **Added knowledge:** Epidemiology of HIV-associated TB by ART status has not been studied in Ethiopia since the implementation of the ‘test-and-treat’ strategy and this study shows that, despite ART rollout, most TB cases among PWH still occur before (or in connection to) ART initiation.
- **Global health impact for policy and action:** This study provides new and useful information to the readers of Global Health Action and the Ministry of Health in Ethiopia and other countries affected by TB and HIV for current and future management of HIV-associated TB.

## Background

People with HIV (PWH) have an overall 20-fold higher risk of tuberculosis (TB) compared to HIV-seronegative individuals, with the major burden of HIV-related TB in sub-Saharan Africa [1,2]. Antiretroviral therapy (ART) confers reduced risk of TB and is considered to be the most important intervention for TB prevention among PWH [3]. The rollout of ART in sub-Saharan Africa is therefore expected to lead to reductions in HIV-related TB. However, TB remains the leading cause of mortality among PWH, and accounts for one-third of HIV-related fatalities globally (214,000 deaths caused by HIV-associated TB in 2020) [4]. The persistence of high mortality linked to TB/HIV coinfection could reflect late diagnosis of HIV, with HIV not detected until disease manifestations, such as TB, have developed. This could also possibly be due to disengagement from HIV care and ART interruption [5]. In a recent report, 46% of TB cases among PWH globally in 2021 occurred in persons not receiving ART [6]. In addition, the risk of TB in PWH receiving ART remains higher than that among HIV-seronegative persons, especially in TB-endemic settings [7]. In a study from Malawi, it was found that the proportions of HIV-associated TB occurring during long-term ART increased over a 10-year period 2008–2017; this finding implies a shift in the epidemiology of HIV-associated TB in settings with high prevalence of both infections [8]. Similar to many countries in sub-Saharan Africa, TB is common among PWH in Ethiopia, with an estimated 20% of all TB cases occurring in PWH [9], and 40% of HIV-related deaths being due to TB [10]. Previous studies from Ethiopia on TB/HIV coinfection have mainly focused on risk factors and characteristics of PWH with TB [11–13], but the proportions of HIV-associated TB with regard to ART status have not been reported.

In this study, we have assessed notifications of TB among PWH in Adama, a city in Central Ethiopia, during an 8-year period in connection with the introduction of the ‘test-and-treat’ strategy for HIV, which recommends immediate ART initiation for all people diagnosed with HIV. Our primary objective was to determine the proportions of TB cases among PWH with regard to ART status at the time of TB diagnosis during this time period.

## Methods

### Setting

This retrospective study of PWH diagnosed with TB was conducted at five public health centers and three hospitals in the city of Adama and the surrounding area in Central Ethiopia (estimated population in the uptake area 1,000,000 people). Adama is located on the highway connecting Ethiopia’s capital Addis Abeba with the Red Sea Coast. This mainly urban area has a higher HIV prevalence than the national average (estimated 1.8%, vs. 0.87%) [14].

**Figure 1.**
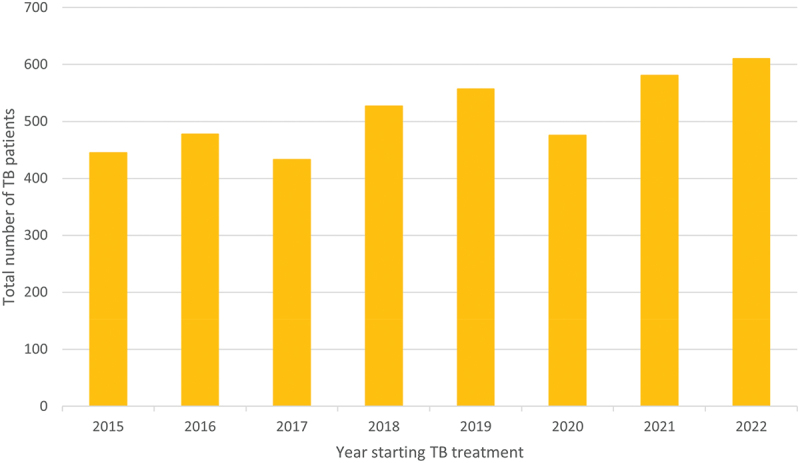
Number of persons treated for TB at the study facilities during the study period.

Abbreviations: TB, tuberculosis.

In this setting, a total of 4,589 persons were registered in the TB registers at the study facilities 2015–2022. The total number of TB cases increased by calendar year (Figure 1).

According to Ethiopian TB/HIV guidelines, regular TB screening in PWH is recommended, based on a clinical algorithm, followed by further targeted investigations, using Xpert MTB/RIF on sputum samples as the initial confirmatory diagnostic test in most situations [10]. Since 2017, Ethiopia has implemented the test-and-treat strategy for HIV, with all PWH eligible to start ART directly after diagnosis [15].

### Study design and participants

Persons diagnosed with HIV and TB (January 2015 to December 2022) were identified from ART registers at the respective facilities. Study data were retrieved from these electronic registers and included age, sex, ART initiation date, TB treatment initiation date and follow-up status at the time of data collection; February–April 2023. The start date of data collections (2015) was chosen because we wanted to follow TB notifications in connection to the introduction of the test-and-treat strategy. Data on the total number of persons receiving TB treatment during the study period were obtained from paper-based TB registers kept at the respective facilities.

We assessed notifications of TB by calendar year during the study period. TB cases were categorized by ART status at the time of TB diagnosis. Since previously unrecognized TB can become clinically manifest due to ART-related immune restoration during the first 6 months of ART (unmasking TB) [16], we considered TB cases notified up to 6 months after starting ART as pre-ART TB (in addition to TB cases notified before ART initiation). Patients with TB diagnosis more than 6 months after starting ART were categorized as ART-associated TB.

We counted one TB episode per patient, with identification of the most recent episode.

### Statistical methods

The total number of PWH with TB were compared by calendar year to determine the trend of notifications and distribution of TB cases among PWH with regard to ART status. Participant demographics were described by age and sex. Age was described using mean and standard deviation. Normality was assessed using both visual and statistical methods. Although the Shapiro-Wilk and Kolmogorov–Smirnov tests indicated significant deviations from normality (*p* < 0.05 for both Pre-ART TB and ART-associated TB groups), these tests are known to be sensitive with large sample sizes, often detecting minor deviations that are not of practical significance.

To provide a more comprehensive assessment, we also examined Q-Q plots, detrended Q-Q plots, and histograms with overlaid normal curves. The Q-Q plots and histograms suggest that the age distributions in both groups were approximately normally distributed, with the data points closely following the reference line in the Q-Q plots and the histograms showing a bell-shaped curve.

Given the above assessments, we concluded that the age data was sufficiently normal for the purposes of our analysis. Therefore, an independent t-test was conducted to compare mean ages between groups. The distribution of sex was evaluated using Pearson’s chi-square test. All analyses were performed using Excel (Microsoft Corp., version 2401) and SPSS (IBM Corp., version 28.0). A p-value <0.05 was considered as statistically significant.

### Ethical considerations

Ethical approval for the study was obtained both from the AHRI/ALERT Ethics Review Committee (AAERC), and the Oromia Regional Health Bureau (both in Addis Ababa, Ethiopia).

## Results

### TB among PWH in the uptake area 2015–2022

During the study period, 8,926 PWH were registered at the study sites (among whom 61.6% were female). In all, 993 (11.1%) had been diagnosed with TB. Data on these individuals are shown in Tables 1 and 2.

**Table 1.** Characteristics of people with HIV and tuberculosis based on ART status at TB diagnosis.

		Total PWH with TB (*n* = 993)	Pre-ART TB^a^ (*n* = 617)	ART-associated TB^b^ (*n* = 376)	p-value
Mean age (SD)		40.0 (11.8)	39.2 (11.7)	41.3 (11.9)	0.006
Sex	Male	531 (53.5%)	336 (54.5%)	195 (51.9%)	0.426
	Female	462 (46.5%)	281 (45.5%)	181 (48.1%)	
Age category					
<15		21 (2.1%)	17 (2.8%)	4 (1.1%)	
15–24		58 (5.8%)	31 (5.0%)	27 (7.2%)	
25–34		215 (21.7%)	150 (24.3%)	65 (17.3%)	
35–44		374 (37.7%)	234 (37.9%)	140 (37.2%)	
45–54		219 (22.1%)	125 (20.3%)	94 (25.0%)	
55–64		84 (8.5%)	48 (7.8%)	36 (9.6%)	
65+		22 (2.2%)	12 (1.9%)	10 (2.7%)	

Abbreviations: PWH, people with HIV; ART, antiretroviral therapy; TB, tuberculosis. Data presented as n (%) if not specified otherwise. P-value derived using Student’s t-test for age and chi2 test for sex. Age is described in years.

^a^TB diagnosis ≤6 months after starting ART.

^b^TB diagnosis >6 months after starting ART.

**Table 2.** Table of number of PWH with TB by time on ART.

		Pre-ART TB^a^			ART-associated TB^b^	
		TB diagnosis before starting ART	TB diagnosis 0-3 months after starting ART	TB diagnosis 3-6 months after starting ART	TB diagnosis 6-12 months after starting ART	TB diagnosis >12 months after starting ART
Total		451 (45.4%)	125 (12,6%)	41 (4,1%)	35 (3,5%)	341 (34,3%)
Mean age (SD)	Male	41.2 (11.6)	41.4 (12.2)	43.3 (12.1)	41.6 (7.7)	43.2 (11.8)
	Female	36.6 (11.2)	37.0 (10.5)	35.5 (9.6)	37.9 (9.8)	39.6 (12.3)
Sex	Male	252 (55.9%)	60 (48.0%)	24 (58.5%)	22 (62.9%)	175 (51.3%)
	Female	199 (44.1%)	65 (52.0%)	17 (41.5%)	13 (37.1%)	166 (48.7%)

Abbreviations: PWH, people with HIV; ART, antiretroviral therapy; TB, tuberculosis.

^a^TB diagnosis ≤6 months after starting ART.

^b^TB diagnosis >6 months after starting ART.

The mean age of individuals with pre-ART TB was 39.2 years [SD 11.7] compared to 41.3 years [SD 11.9] for those with ART-associated TB (*p* = 0.006). The proportion of men was similar at 54.5% and 52.0% for pre-ART TB and ART-associated TB, respectively (*p* = 0.426) (Table 1).

Among 617 persons with pre-ART TB, 451 (73.1%) had been diagnosed before starting ART, whereas 125 (20.3%) were diagnosed within the first three months of ART, and 41 (6.6%) between three and six months after starting ART.

Median time from ART initiation to TB diagnosis among ART-associated TB was 6.0 years [interquartile range 2.7–9.6].

### Distribution of TB notifications among PWH by ART status

**Figure 2.**
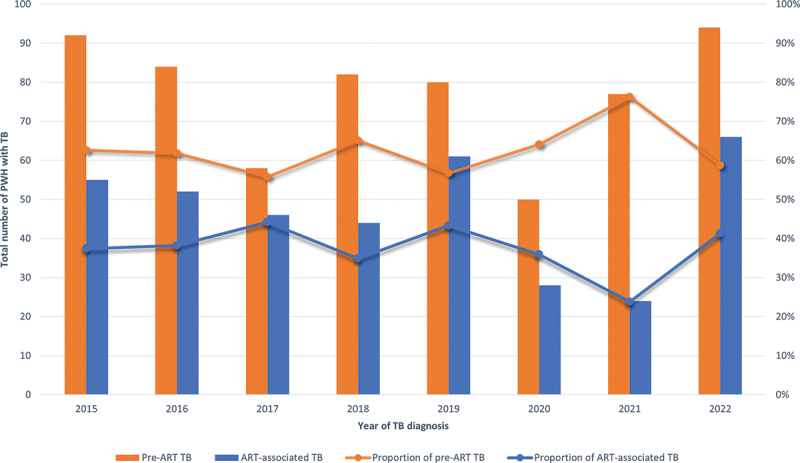
Distribution and proportion of total number of TB cases by ART status and by calendar year of TB diagnosis.

Abbreviations: PWH, people with HIV; ART, antiretroviral therapy; TB, tuberculosis.

The annual number of PWH with TB remained relatively stable over time during the study period, with a mean of 124 TB notifications per year (Figure 2).

For the whole study period (2015–2022), the mean annual number of pre-ART TB notifications was 77 (peaked at 94 cases 2022 and was lowest at 50 cases 2020), compared to 47 for ART-associated TB cases (peaked at 66 cases 2022 and was lowest at 24 2021), with ART-associated TB cases constituting a mean of 37.4% (range 23.8%–44.2%) of all TB cases among PWH annually (Table 3; Figure 2).

**Table 3.** Table of proportions of PWH with TB by time on ART by calendar year.

	2015	2016	2017	2018	2019	2020	2021	2022
Proportion of pre-ART TB	62.6%	61.8%	55.8%	65.1%	56.7%	64.1%	76.2%	58.7%
Proportion of ART-associated TB	37.4%	38.2%	44.2%	34.9%	43.3%	35.9%	23.8%	41.3%

Abbreviations: ART, antiretroviral therapy; TB, tuberculosis.

## Discussion

In this study, covering an 8-year period 2015–2022 in connection to the introduction of the ‘test-and-treat’ strategy for HIV in Ethiopia, the annual number of TB notifications remained relatively stable among PWH in this mainly urban area. Furthermore, most cases of HIV-associated TB occurred before or in connection to ART initiation throughout the study period.

Our findings contrast with those of a nationwide study conducted in Malawi, in which declining TB incidence in PWH 2008–2017 was observed, along with increasing proportions of TB cases occurring in long-term ART recipients [8]. In our study population, the majority of TB cases were detected in connection to HIV diagnosis. We could not determine whether HIV had been diagnosed as part of provider-initiated testing at TB clinics; yet, it is clear that HIV was detected at advanced disease stages in these individuals. This phenomenon, known as late presentation, remains common, both in low-and high-income settings, and has been shown to account for a major proportion of HIV-related TB cases [17]. In our study population, the proportion of men was greater among PWH with TB, irrespective of ART status. The increased incidence of TB among men is well known and may be due to both sex- and gender-related factors, including higher risk of TB exposure, as well as characteristics associated with TB disease progression [18]. Furthermore, late HIV diagnosis has been found to be more common among men [19]. Our findings emphasize the importance of further scale-up of HIV testing in Ethiopia, which needs to be adapted to populations at particularly high risk of HIV-related TB and linked to client-friendly HIV care [20].

Among pre-ART TB cases in our study, 73.1% had been diagnosed before starting ART. We also categorized TB cases occurring within six months after starting ART as pre-ART TB, since such cases are likely to represent TB disease present at the time of ART initiation missed due to lack of clinical manifestations – so called unmasking TB [21]. This phenomenon may explain the paradoxically elevated incidence of TB during this early period of ART, illustrating the difficulty to reliably exclude TB in PWH [16]. Our findings are in line with those reported from Thailand by Suwanpimolkul et al. [22], showing high TB incidence during the first year after starting ART, in particular during the first three months, whereas TB incidence during long-term ART declined over time and was comparable to the general population after ten years of ART. Furthermore, sub-clinical TB, which is especially common in persons with advanced immunosuppression at HIV diagnosis, should also be considered with regard to initiation of tuberculosis preventive therapy (TPT). Early TPT is strongly recommended in people starting ART but could lead to inadvertent inadequate TB therapy in persons with TB disease, with delayed accurate diagnosis in such individuals and potential for emergence of TB drug resistance [23]. According to Ethiopian guidelines, TPT should be administered at enrolment to HIV care after TB has been ruled out [10]. However, similar to other low-income countries, low rates of TPT completion among PWH in Ethiopia have been reported (62.1% in a recent study conducted in the Tigray region) [24].

The uptake area where this study was performed is characterized by high population mobility and urbanization. Similar to many other cities in Ethiopia, HIV prevalence in Adama is higher than the national average [14]. Data on overall TB notifications in the study sites in this urban area show the same pattern as that observed among PWH, without decreasing trends, a finding which is in contrast to national TB reports [25]. The persistent high number of TB notifications could be due to the introduction of better diagnostic methods for TB case-finding (in particular, GeneXpert technology which has replaced previous microscopy-based diagnostic algorithms [26]. Interestingly, a previous study from Adama showed that a considerable proportion of people treated for TB in that city originated from other areas, which could result in exaggerated numbers of TB notifications attributable to the uptake area [27]. Yet, it is not likely that this would influence notification trends during the study period. Importantly, the stable number of TB notifications could reflect continuous TB exposure in the community, as suggested by a study on TB infection among women of reproductive age conducted in Adama, which indicated a 2.1% annual rate of TB acquisition in this population [28].

Similar to reports from other countries, we observed a decrease of TB notifications in 2020, both among PWH and in the overall population, which is likely to be related to changes in healthcare services during the COVID-19 pandemic. The restrictions imposed during this period have also been associated with increased TB mortality [29], yet such analysis was beyond the scope of the current study.

Although the majority of TB cases among PWH belonged to the pre-ART category throughout the study period, considerable numbers of long-term ART recipients were diagnosed with TB; indeed, this number increased from 24 in 2021 to 66 in 2022, which might indicate an increase in ART-associated TB in the study population. Our study was not designed to investigate factors associated with incident TB during ART, such as lack of viral suppression and persistent CD4 lymphopenia, that have been identified as risk factors for ART-associated TB in previous studies [30]. Furthermore, we could not assess the excess risk of TB among PWH receiving long-term ART compared to HIV-seronegative persons living in the uptake area. Future studies of these issues are warranted.

To our knowledge, this study is the first from Ethiopia on the epidemiology of HIV-associated TB since the introduction of the ‘test-and-treat’ strategy, investigating TB among PWH by ART status. The study was performed in an urban area with high HIV prevalence and high TB incidence, covering all public health facilities providing HIV care.

Our study has certain limitations. First, since the study was conducted in a limited uptake area, the results may not be representative for Ethiopia on a national level, nor for other urban areas in the country. Second, some PWH may have received TB treatment in other health facilities than those included in our study, and information on TB diagnoses in such cases may not have been reported to the ART clinics. This may have led to an underestimation of TB notifications among PWH; however, this phenomenon would not change our main finding of a lack of annual decrease TB notifications in the study population. Third, the database did not allow for entry of several cases of TB in individual patients. This could have underestimated the true number of TB notifications during the study period but would not influence our finding of persistently high occurrence of TB among PWH during the study period. Fourth, we lacked data on factors that may influence the risk and presentation of TB disease in PWH, such as type of TB, history of previous TB and CD4 count. However, our study was not designed to analyze risk factors or the excess risk of TB in PWH receiving ART since our objective was to analyze proportions of TB in PWH regarding ART status. Fifth, analyses regarding incidence and rates per capita of TB in PWH were not part of our objective. We estimated the number of PWH to have been relatively stable during the study period, therefore basing our analysis on the number of cases and not rates per capita.

## Conclusion

In this mainly urban uptake area in Central Ethiopia, we found persistently high numbers of TB notifications among PWH during 2015–2022 in connection to introduction of the ‘test-and-treat’ strategy for HIV. Throughout the study period, most cases of HIV-associated TB were diagnosed before or within 6 months after ART initiation, implying late diagnosis of HIV in a considerable proportion of PWH.
